# User Interactions With Health Insurance Decision Aids: User Study With Retrospective Think-Aloud Interviews

**DOI:** 10.2196/27628

**Published:** 2021-10-26

**Authors:** Wayne C W Giang, Emma Bland, Jeffrey Chen, Coralys M Colón-Morales, Michelle M Alvarado

**Affiliations:** 1 Department of Industrial and Systems Engineering University of Florida Gainesville, FL United States

**Keywords:** insurance, health, health benefits plans, employee, decision aids, cognitive ergonomics, human factors engineering

## Abstract

**Background:**

Two barriers to effective enrollment decisions are low health insurance literacy and lack of knowledge about how to choose a plan. To remedy these issues, digital decision aids have been used to increase the knowledge of plan options and to guide the decision process. Previous research has shown that the way information is presented in a decision aid can impact consumer choice, and existing health insurance decision aids vary in their design, content, and layout. Commercial virtual benefits counselors (VBCs) are digital decision aids that provide decision support by mimicking the guidance provided by an in-person human resources (HR) counselor, whereas more traditional HR websites provide information that requires self-directed navigation through the system. However, few studies have compared how decision processes are impacted by these different methods of providing information.

**Objective:**

This study aims to examine how individuals interact with two different types of health insurance decision aids (*guided* VBCs that mimic conversations with a real HR counselor and *self-directed* HR websites that provide a broad range of detailed information) to make employer-provided health insurance decisions.

**Methods:**

In total, 16 employees from a local state university completed a user study in which they made mock employer-provided health insurance decisions using 1 of 2 systems (VBC vs HR website). Participants took part in a retrospective think-aloud interview, cued using eye-tracking data to understand decision aid interactions. In addition, pre- and postexperiment measures of literacy and knowledge and decision conflict and usability of the system were also examined.

**Results:**

Both the VBC and HR website had positive benefits for health insurance knowledge and literacy. Previous health insurance knowledge also impacted how individuals used decision aids. Individuals who scored lower on the pre-experiment knowledge test focused on different decision factors and were more conflicted about their final enrollment decisions than those with higher knowledge test scores. Although both decision aids resulted in similar changes in the Health Insurance Literacy Measure and knowledge test scores, perceived usability differed. Website navigation was not intuitive, and it took longer to locate information, although users appreciated that it had more details; the VBC website was easier to use but had limited information. Lower knowledge participants, in particular, found the website to be less useful and harder to use than those with higher health insurance knowledge. Finally, out-of-pocket cost estimation tools can lead to confusion when they do not highlight the factors that contribute to the cost estimate.

**Conclusions:**

This study showed that health insurance decision aids help individuals improve their confidence in selecting and using health insurance plans. However, previous health insurance knowledge plays a significant role in how users interact with and benefit from decision aids, even when information is presented in different formats.

## Introduction

### Background

Health insurance enrollment is a complex decision based on many factors, such as price, product attributes, and current health status, and can significantly impact a person’s health and financial circumstances [1]. Making an informed decision depends on a person’s knowledge, literacy, cognitive skills, and confidence to carry out said tasks [2]. Despite the consequences of this choice, only 4% of adults in the United States understand basic health insurance terminology and often get overwhelmed by the complexity of the decision [3,4]. In addition, health insurance plans can be challenging to understand, especially if the decision-maker has limited financial or health insurance literacy [2,5]. Previous research has shown that the way information is presented in the decision aid can impact consumer choice [6]. Factors such as the order in which plans are presented, word choice and symbol use, and difficulty in finding information can significantly affect trust in the information and consumer choice [1,7]. Therefore, understanding how individuals interact with sources of health insurance information is a key component in improving informed decision-making.

Over 55% of Americans receive health insurance from their employer [8]. Some employers have used virtual benefits counselors (VBCs) to provide further decision support for their employees. VBCs are designed to provide guided support by mimicking a one-on-one interaction with a human resources (HR) representative. Although there has been limited research on health insurance decision aids [6,9,10], VBCs are a relatively new product for supporting health insurance choices that combine access to tools, such as cost estimators, and further guidance and recommendations presented through a conversational interface. The effects of this more guided approach to decision-making are still not well understood, and little research has examined how guided systems affect consumer health insurance decision-making when compared with traditional self-directed methods such as websites.

VBCs may be of particular benefit to low-literacy consumers, as previous research has shown that these consumers often confuse health insurance concepts [2]. Kodagoda et al [11] found that users with low reading literacy, numeracy, and digital literacy tend to end information searches early (due to perceived completion of task), take longer to complete the tasks, and have less directed searching strategies than high-literacy users. In addition, low-literacy users are less able to predict where information would be on a website accurately, are less able to find information on websites, and are less likely to verify the information found. VBCs guide users through the enrollment process and provide relevant information and recommendations on the basis of cost calculations using a conversational interface. Consequently, they have the potential to help users, especially those with lower literacy, make informed health insurance enrollment decisions. The guided decision support provided by a VBC system has the potential to improve a user’s ability to find relevant information and ensure that they consider important factors while making their enrollment decisions.

User interactions with health insurance information and digital decision aids, such as VBCs and HR websites, are likely to be impacted by their incoming knowledge and previous health insurance use [3]. Furthermore, if participants are able to become more knowledgeable and literate about health insurance information, they are likely to become more informed and confident decision-makers. These changes may have influenced the factors considered during the decision process. However, few studies have directly compared VBCs and HR websites, particularly for employer-provided plans. Most studies have also largely relied on reviews of health insurance enrollment data sets [12] or web-based evaluations of decision aids [10] to evaluate the effectiveness of these decision aids, which makes it more difficult to understand the user’s decision process as they interact with these tools. Thus, this exploratory study uses a think-aloud method to understand how an individual’s interaction with the guided VBC decision aid versus self-directed information provided on HR websites influences the user’s decision process and measures that may impact the final decision quality: health insurance knowledge, literacy, decision conflict, system usability, and decision processes.

### Health Insurance Decision Aids

#### Virtual Benefits Counselors

**Figure 1:**
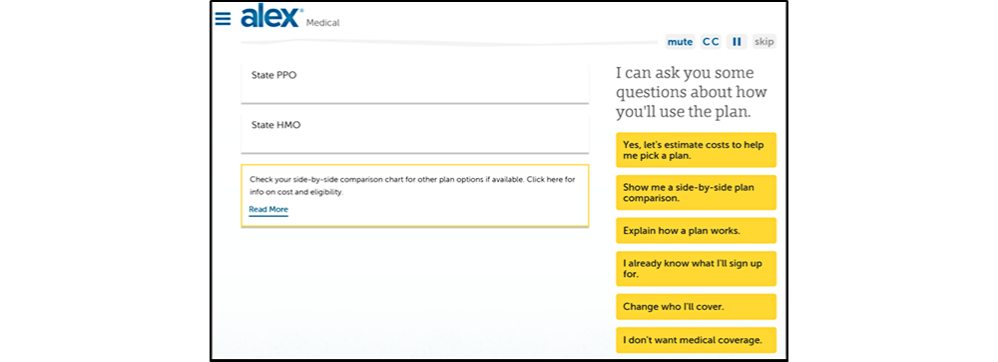
Screenshot of the Jellyvision Lab Inc’s Alex interface.

This study uses Alex, a VBC created by Jellyvision Lab Inc, which was customized to the specific plans provided by the employer. Alex uses a conversational question-and-answer interface with colorful animations, text, and a fully voiced personality (Figure 1). The conversation guides the interactions of the user and helps to structure the decision process. Alex also interjects at different points to provide definitions or clarifications of the information provided.

#### HR Website

HR benefits websites provide a self-directed experience in which users navigate freely between different pages. During the study, the state university’s HR benefits website had health information distributed across two areas: a general benefits section and a dedicated section on health insurance. Information about eligibility, comparison charts between plans, and enrollment processes can be found on these pages. The HR website also provides links to the state’s health insurance website, where details about the different plan options (Health Maintenance Organization and Preferred Provider Organization) including costs (ie, deductibles, premiums, copays, and coinsurance), network size, and coverage were presented using digital brochures and tables. Overall, the website provided detailed information that was distributed nonlinearly across multiple pages and lacked the cost estimation tools found in the VBC.

## Methods

### Participants

Participants were recruited as part of a survey studying sources of health insurance information used to make enrollment decisions at a local employer (a university campus) that has been reported elsewhere [13,14]. Links to the survey were distributed to the staff and faculty, resulting in a total of 140 complete responses. Of these 140 responses, 113 (80.7%) indicated an interest in participating and were contacted for recruitment. A total of 16 employees enrolled and completed the user study and were randomly assigned to either the VBC or HR website. All participants indicated that they had primary (11/16, 69%) or shared responsibility (5/16, 31%) for health care decisions in their household. Data collection was impacted by COVID-19 during the data collection phase, which resulted in a smaller participant sample than that initially planned. This study was approved by the local institutional review board.

### Experimental Task

To understand how guided and self-directed information support affects informed health insurance decision-making, a *mock health insurance enrollment task* along with a *retrospective think-aloud method* was used. Retrospective think-alouds can detect issues during user interactions and help encourage participants to verbalize comments about their thoughts and interactions with the system [15].

#### Mock Health Insurance Enrollment Task

Participants were asked to make a mock health insurance enrollment decision for the upcoming year for their household. They were provided with a decision aid to assist them with this task: either the VBC or HR website. Participants were asked to stay within the bounds of the provided system and were directed to return to the system if they exceeded these bounds (eg, left the HR website to use Google).

#### Think-Aloud Interview

A retrospective think-aloud method was used to understand the participants’ motivations and strategies when navigating through their assigned system. Participants were asked to explain their thought process, the information they were looking for, and anything they were confused or unsure about while watching a video of their gaze behavior while using the system, which was captured using an eye tracker. The experimenters occasionally prompted participants to verbalize their thoughts and reasoning behind decisions throughout the interview and ask for clarifications when required. The interviews were recorded and transcribed using a Health Insurance Portability and Accountability Act–compliant transcription service.

### Procedure

The experiment consisted of five phases. (1) *Training*: participants were introduced to the purpose of the study, eye-tracking equipment, and think-aloud methodology. They then went through a training session where they were familiarized with the eye tracking and think-aloud process with a simple decision task. (2) *Pre-experiment questionnaire*: participants were given a pre-experiment questionnaire that measured their health insurance literacy and health insurance knowledge; (3) *mock health insurance enrollment task,* as described in the experimental tasks. (4) *Postexperiment questionnaire*: after making their enrollment decision, participants were again asked about their health insurance literacy and knowledge. Participants were also asked to fill a decision conflict scale and to rate the usefulness and ease of use of the system; (5) *a retrospective think-aloud interview,* as described in the experimental tasks*.* The experiment was conducted by a trained graduate and undergraduate research assistant in an office-like environment. The experiment took approximately 90 minutes.

### Experimental Design and Measures

The main independent variable was the decision aid system used to assist with the mock enrollment decision, either the VBC or HR website. The response measures were the thematic analysis of the think-aloud interviews and questionnaire data delivered before and after engaging with the system.

The thematic analysis of the think-aloud interviews allowed for the identification of *decision factors* discussed by participants during their use of the 2 systems. These factors provide insight into the variables considered by the participants while making enrollment decisions. Think-aloud interviews were also used to identify themes about *how participants interacted* with the guided VBC and self-directed HR website decision aid systems.

Four sets of questionnaire data were also examined to help understand the participants’ health insurance literacy, confidence in decision-making, and perceived usability of the 2 systems.

The *Health Insurance Literacy Measure (HILM)* is a 21-item self-report questionnaire that asks participants to assess their self-efficacy in four subcomponents of health insurance related to confidence and likelihood of demonstrating health insurance literate behaviors: *confidence in choosing a plan*, *comparing plans, confidence in using a plan*, and *being proactive* when using a plan. Participants rated each item on a 7-point scale (from “1-extremely low/extremely unlikely” to “7-extremely high/extremely likely”), which was averaged to calculate a score for each category.

The *knowledge tests* included seven true or false questions about different health insurance concepts and definitions. One test was adapted from a previous study on health insurance decision aids by Politi et al [10]. A second version of the test was created with a similar difficulty. The order of the tests was counterbalanced.

The *SURE* (Sure of myself; Understand information; Risk-benefit ratio; Encouragement) *measure* is a series of 4 yes or no questions designed to measure decisional conflict, with higher scores indicating less decisional conflict [16].

Participants were also asked to rate the *usefulness* and *ease of use* of the system on a 10-point scale (from “1-not useful at all/not easy to use at all” to “10-extremely useful/extremely easy to use”).

### Apparatus

Participants used a 15-inch laptop with an attached mouse to navigate through the decision aids. Eye-tracking data were collected using a Tobii Pro Nano screen-based eye tracker, and a retrospective think-aloud was facilitated using Tobii Pro Lab software [17].

### Data Analysis

Emergent Themes Analysis (ETA) was used to identify the factors that each participant mentioned during their enrollment decision while using the 2 decision aid systems. ETA has previously been used to understand decision processes [18,19] and user interactions with decision aids [11,20]. The process started with identifying broad themes or conceptually related topics found within the transcripts through an initial high-level reading of the data. Three researchers (WCWG, JC, and MMA) completed this process and identified a number of common themes that were mentioned by many of the 16 participants. These themes were consolidated through a card-sort. This analysis was supplemented with observations and quotations about the users’ strategies to engage with the decision aids. Participants were also divided into 2 groups—those who came into the experiment with lower health insurance knowledge (scores<6/7) and those with higher health insurance knowledge, and this variable was used in subsequent analyses.

Owing to the small sample size, exploratory data analysis was conducted on the questionnaire data to better understand the effects of interacting with the decision aids on health insurance literacy and knowledge, SURE scores, and usefulness and ease of use ratings. These descriptive quantitative data were further supported using excerpts from think-aloud interviews.

Data from 2 participants were partially impacted by data recording issues. The pre-experiment questionnaire data for 1 VBC participant was lost, and their data were excluded from the analysis of the HILM, knowledge test, SURE scores, and usefulness and ease of use ratings. An HR website participant had eye-tracking data recording issues during the mock health insurance enrollment task, resulting in a think-aloud interview based on a video of the interactions rather than prompted by eye-tracking data; these data were kept within the data set.

## Results

### Respondent Demographics and Characteristics

The demographics of the 16 participants are presented in Table 1. Participants were predominantly female (12/16, 75%), and the majority were married or in a domestic partnership (10/16, 63%). However, most of the participants came from small households of either 1 or 2 individuals (11/16, 69%). Finally, most participants chose the Health Maintenance Organization plan (10/16, 63%) over the Preferred Provider Organization plan. Both plans had similar desirability as they covered similar procedures and services and had the same monthly premium but differed in terms of network, deductible, and coinsurance or copays.

**Table 1:** Participant demographics and plan choice.

Demographics			VBC^a^ participants (n=8), n (%)		Human resources website participants (n=8), n (%)		Total (n=16), n (%)
**Gender**							
	Female	7 (88)		5 (63)		12 (75)	
	Male	1 (12)		3 (37)		4 (25)	
**Age (years)**							
	18-24	1 (12)		1 (12)		2 (12)	
	25-34	4 (50)		3 (37)		7 (44)	
	35-44	2 (25)		2 (25)		4 (25)	
	45-54	1 (12)		1 (12)		2 (12)	
	55-66	0 (0)		1 (12)		1 (6)	
**Marital status**							
	Married	4 (50)		6 (75)		10 (63)	
	Not married	4 (50)		2 (25)		6 (37)	
**Average time since hire (years)**							
	<1	2 (25)		2 (25)		4 (25)	
	2-5	4 (50)		3 (38)		7 (44)	
	>5	2 (25)		2 (25)		4 (25)	
**Number of additional family members covered in the plan**							
	0	3 (38)		2 (25)		5 (31)	
	1	3 (38)		3 (38)		6 (38)	
	2-4	2 (25)		3 (38)		5 (31)	
**Selected plan**							
	HMO^b^	6 (75)		4 (50)		10 (63)	
	PPO^c^	2 (25)		4 (50)		6 (37)	

^a^VBC: virtual benefits counselor.

^b^HMO: Health Maintenance Organization.

^c^PPO: Preferred Provider Organization.

### Health Insurance Literacy and Knowledge

**Figure 2:**
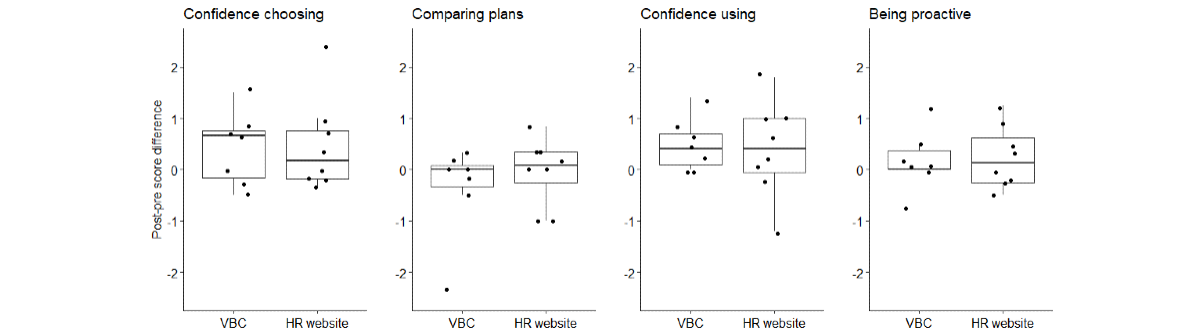
Differences in post- and pre-experiment Health Insurance Literacy Measure scores. HR: human resources; VBC: virtual benefits counselor.

Table 2 shows the pre- and postexperiment scores for the 4 dimensions of the HILM and the knowledge test for the VBC and the HR website. Across the sample, participants tended to rate their confidence and likelihood of performing health insurance literate behaviors as higher than neutral, with the scales relating to likelihood of performing health literate behaviors (eg, comparing plans or being proactive) scoring higher than the confidence scales (eg, confidence in choosing or confidence in using). Figure 2 shows the differences in the HILM scores for each participant after interacting with the decision aid. Across all four subcomponents, the differences appeared to be similar for the VBC and the HR website. However, most participants reported an increase in their confidence in choosing (8/15, 53%) and using (10/15, 67%) their plans, which agreed with the larger magnitude in scores seen in Table 2 (Choosing: Δ=0.43; Using: Δ=0.44). In contrast, the 2 HILMs related to health insurance literate behaviors had more variable results after interaction with both the VBC and the HR website.

Surprisingly, the trend of the data suggested that the *comparing plans* literacy subcomponent decreased after interacting with the decision aids, and the magnitude of this decrease was larger for the VBC. The interview data provided additional evidence for this, with multiple VBC participants commenting that they were often confused by the outputs of the cost estimation tool as the Health Maintenance Organization and Preferred Provider Organization plans resulted in very similar out-of-pocket costs. Thus, participants felt that the VBC recommendations were not useful because they did not understand why a plan was selected. For instance, 1 VBC participant commented:

And this is when I came up to the not very helpful conclusion that the plans are essentially the same. [...] I was a little surprised. I figured there would be a little bit more difference between the two programs. And then, I said, “It’s not really helpful,” because it’s not, I mean, they’re so similar and they don’t do a good job in this [VBC] system, in my opinion, of explaining what the differences are and really highlighting those differences, because there are differences, and I think they really should emphasize those instead of basically saying, “Oh, well, they’re the same.”

[ID03, VBC]

This quote highlights that participants may not have been using the information provided by the VBC’s cost estimation tools to their full extent. As mentioned previously, the cost estimation tool allows users to estimate their yearly out-of-pocket costs by guiding users by estimating health insurance use throughout the year and automatically calculating the final costs for the different plan options. One method for using such a tool is to allow users to estimate the effects of different health insurance use scenarios. For example, participants may want to see how the costs of each plan change if they require major surgery in the upcoming year. Participants appeared to be aware of this possibility, with one participant commenting during their interactions with the cost estimation tool, “But I think, yes of course you want to include like at least one [specialist visit], because at least maybe show you the cost differential.” [ID12, VBC]

By exploring these different outcomes, users may be better able to understand the differences between the provided plans and how uncertainty in their own health insurance use might impact the overall costs. However, none of the participants in our VBC condition used the cost estimation tool. Instead, some participants commented that they would have liked to see how their use choices influenced the VBC’s cost estimation:

That’s exactly what I would have liked, is a breakdown of all those things that I chose, like, which one of them [was contributing to the costs] because then I could be like, “Well, I put two ER costs, but that’s not really the biggest deal here.” I think that would have been helpful.

ID08, VBC

These results suggest that cost estimation tools should highlight how changes in health insurance use would impact the final out-of-pocket costs, and this design change may lead to users considering a more diverse set of health care use scenarios.

As shown in Table 2, participants in the sample had relatively high knowledge test scores (out of 7) before interacting with the decision aids (VBC: median 6, range 3-7; HR website: median 5.5, range 4-7). Their knowledge test scores increased after interacting with the decision aids (VBC: median 6, range 4-7; HR website: median 7, range 6-7). The average improvement in knowledge test scores was similar between the VBC (Δ=1.14) and HR website (Δ=1.13). These results suggest that both decision aid systems improve health insurance knowledge.

**Table 2:** Sample means and SDs for pre- and postexperiment Health Insurance Literacy Measure and knowledge test scores.

Dimensions and test	VBC^a^ (n=7), mean (SD)			Human resources website (n=8), mean (SD)	
	Pre-experiment	Postexperiment	Pre-experiment		Postexperiment
Confidence in choosing	4.10 (1.14)	4.5 (1.03)	4.56 (1.37)		5.02 (1.36)
Comparing plans	5.60 (1.14)	5.24 (1.62)	5.52 (1.40)		5.48 (1.27)
Confidence in using	3.97 (1.23)	4.46 (0.97)	4.45 (1.65)		4.85 (1.61)
Being proactive	5.54 (0.87)	5.71 (0.81)	6.09 (0.76)		6.34 (0.33)
Knowledge test (%)	71.4 (23.2)	87.7 (15.3)	80.3 (15.1)		96.4 (6.6)

^a^VBC: virtual benefits counselor.

### Decision Factors

The thematic analysis in Table 3 identifies several decision factors considered by the participants during their enrollment process. The types of factors considered by participants in both the VBC and HR website conditions were relatively similar, with network size, use costs, and coverage costs being the factors considered most frequently, and the ease of use and understanding plans being the factors considered least frequently.

**Table 3:** Decision factors considered by participants during the enrollment process compared across decision aid (virtual benefits counselor [VBC] vs human resources [HR] website) and pre-experiment knowledge test (KT) scores (low vs high).

Decision factor	Definition	Example quotes	VBC (n=8), n (%)	HR website (n=8), n (%)	Low KT score (n=7), n (%)	High KT score (n=8), n (%)
Network size	Consideration of the size of the network or whether their providers or specialists were within the network	“HMO is not a good thing. I mean, it’s not good. I knew it, but based on your information, again, it’s not good for out of network at all. But still there’s a high probability that I will be out of network, so I want to have some coverage for that specific thing.” [ID01, VBC]	6 (75)	7 (88)	6 (86)	6 (75)
Premium costs	The monthly premium costs for each plan	“Those [premiums] are really the costs that I care about more, because if I’m going to be paying it [...] every single month I’ll want to know that.” [ID06, VBC]	5 (63)	6 (75)	5 (71)	5 (63)
Use costs	Copays, coinsurance, and deductible costs	“It’s talking about the lower deductible at $250 a person or $500 a family.” [ID07, HR website]	6 (75)	7 (88)	4 (57)	8 (100)
Coverage costs	Costs associated with specific treatments, procedures, or care and whether they are covered by the plan	“Generally, I have drug costs [...] so, now I went into the plan because I wanted to see if there was anything on the cost of specialty drugs. [...] Everything I was reading about confirmed that it was the right type of drug [...] it just didn’t tell me the cost. I’m doing the same thing here looking for the cost of the specialty drug, which is 100% covered under the HMO.” [ID15, HR website]	7 (88)	6 (75)	5 (71)	7 (88)
Estimated out-of-pocket costs	The total yearly estimated out-of-pocket costs	“What I was trying to do overall was figure out my total cost for the year in each plan. So then, I had to figure out $500 a year and that’s when I did the premium $420 versus $50 like what the $35 a month savings was. The high-deductible that was again like - the savings would be not enough because the out-of-pocket max is so high.” [ID15, HR website]	6 (75)	6 (75)	6 (86)	5 (63)
Ease of use of plan	How easy it was to use the plan to access and pay for care	“I’m just more familiar with the HMO and all of our – we haven’t had an issue where we couldn’t really find a provider that was under a plan because, you know, should you have chances, it’s pretty – yeah, it’s pretty accessible.” [ID16, VBC]	3 (38)	3 (38)	2 (29)	4 (50)
Ease of understanding the plan	How easy it was to understand the plan	“It seems to me that the HMO is, you know, easier to understand. I tend, you know, not to trust… these two [Plan Name] plans, because it is confusing.” [ID02, HR website]	2 (25)	2 (25)	3 (43)	1 (13)

These results were surprising because of the very different information presentation methods and tools provided in the guided VBC and self-directed HR website. The VBC explicitly offers a series of factors to consider through cost estimation tools and highlights important plan features in comparison tables. In contrast, the HR website requires individuals to identify and seek relevant information and tools. The fact that participants using both systems were able to identify similar types of decision factors suggests that participants in this study had some idea of what elements they should look at while searching for health insurance and agreed with the HILM and knowledge test scores discussed previously. They did not necessarily depend on the guidance provided by the VBC to identify new, personally significant decision factors.

However, the ETA helped identify 2 elements that might make it difficult for participants to use the decision factors, even though they had identified them. First, participants may struggle to obtain accurate information about each factor; either they were not able to quickly locate the information in the system (a common complaint for the HR website condition because of the layout of the pages or not knowing the correct keyword to search for, eg, “My routine bloodwork, I have a hard time kind of finding bloodwork, but eventually, I figure out what term to search for. I just used a search function to figure out both of the keywords that I had in my mind” [ID05, HR website]) or the information was not provided at a level of detail that the participant desired (a common issue in the VBC condition, see the coverage cost quote in Table 3 for a contrasting HR website example). Second, participants may not know how to use the identified factors to make a final decision. The VBC benefited from a cost estimation tool that helped users estimate their yearly costs based on user-provided estimates of their health insurance use for the coming year and calculate a final value. However, participants in the HR website condition were required to perform these calculations. Although many of the participants in the HR website condition attempted to do so (eg, estimated out-of-pocket cost quote in Table 3), these calculations are likely to be more difficult and more error-prone without decision support.

Table 3 also shows which factors participants with higher and lower prior knowledge discussed. Many participants with higher pre-experiment knowledge test scores mentioned use costs (eg, copays, coinsurance, and deductibles) during their think-aloud interviews and considered ease of plan use during their decision-making process. In contrast, participants with lower knowledge test scores discussed how easy it was to understand a health care plan during the think-aloud interviews. These results further emphasize the vital role of health insurance literacy in how individuals interact with decision aids, regardless of the guided or self-directed presentation methods.

### Decision Conflict

The SURE score represents the amount of decision conflict that a decision-maker has about their final choice. Any score below 4 indicated a conflict or lack of comfort with the final decision. As seen in Table 4, participants in both the VBC and HR website conditions had some decision conflict, although those in the HR website condition appeared to have less conflict. This was surprising because the VBC provided explicit guidance for the decision process, whereas the HR website did not. However, 1 participant stated that it was this additional guidance that made them less confident in their final decision when speaking about the VBC’s cost estimation tool:

Because it makes me have to stop and think about these estimates of how much care I think I would need [...] versus if I was just looking at the flat numbers in my head, I probably wouldn’t say, “Oh, how many of these visits do you do?” [...] I just look at the flat numbers and say, “Well, this one is cheaper.” And I go with that.

[ID12, VBC]

**Table 4:** SURE (Sure of myself, Understand information, Risk-benefit ratio, Encouragement) scores for system type (virtual benefits counselor [VBC] vs human resources [HR] website) and lower versus higher preinteraction knowledge test (KT) scores.

SURE score	VBC (n=7), n (%)	HR website (n=8), n (%)	Lower KT scores (n=7), n (%)	Higher KT scores (n=8), n (%)
0	2 (29)	0 (0)	2 (29)	0 (0)
1	0 (0)	2 (25)	2 (29)	0 (0)
2	1 (14)	1 (13)	0 (0)	2 (25)
3	1 (14)	0 (0)	1 (14)	0 (0)
4	3 (43)	5 (63)	2 (29)	6 (75)

In contrast, participants in the HR website condition may have considered fewer unknown situations or novel factors when making their decision. Many of the decision factors considered by the HR website participants were those that had already been previously considered before interacting with the decision aid or an ongoing medical condition, for example:

Like as my [spouse] and I get older, [spouse] is needing some surgeries. My children are in sports. We travel quite a bit for sports. You just never know when someone is going to get hurt.

ID07, HR website

As these factors were produced by the participants on their own volition, they may have resulted in less conflict when making the decision.

The SURE scores of participants who had lower pre-existing knowledge (eg, lower scores on the pre-experiment knowledge test) also appeared to have more decision conflict than those who had higher scores (Table 4). Similar to participants in the HR website condition, participants who had higher pre-existing knowledge also tended to have specific factors that they searched for during the decision process, regardless of the system (eg, “I did choose to look at the out-of-network versus the in-network because it’s kinda topical right now for me. So, my [spouse] is gonna go to New Mexico for a two – like, two plus months [...] and [they] currently got some health problems.” [ID16, VBC]).

Participants with higher knowledge also tended to be more active users of their current plans and were much more familiar with the health insurance enrollment decision. Thus, regardless of the system type, previous knowledge plays a significant role in confidence in their final decision.

### Usefulness and Ease of Use

**Figure 3:**
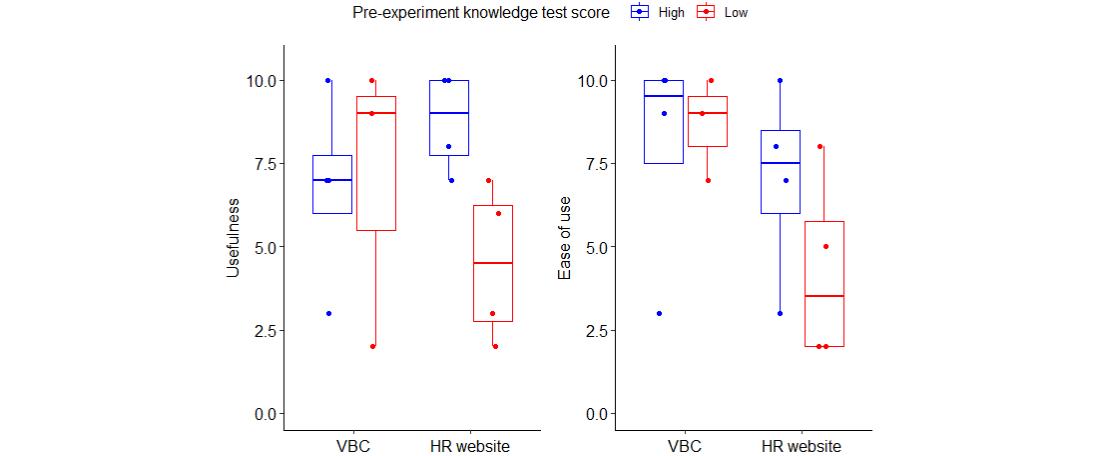
Participant ratings of usefulness and ease of use across system and pre-experiment knowledge test scores. HR: human resources; VBC: virtual benefits counselor.

Finally, participants were asked to rate the usefulness and ease of using the two systems. Figure 3 shows the ratings across the two systems for participants who scored low and high on the pre-experiment knowledge test. The sample data suggest that pre-existing knowledge about health insurance played a role in how the participants rated the usability of the VBC and HR website. A number of participants stated that they believed that the VBC would be useful for those who had lower health insurance literacy (eg, “I mean I’m familiar a little bit with insurance like I definitely am not a person who knows nothing. So, I can see at how it can be helpful for somebody with no knowledge of health care.” [ID16, VBC]), whereas those with higher pre-existing knowledge commented that the VBC did not have all the required details (eg, the VBC was “not tied in. I still have to go to these crazy insurance websites, actually get to the information, where I really want to be at that detail I want.” [ID12, VBC]). This trend was supported by the usefulness ratings with lower knowledge participants rating slightly higher usefulness than those with higher knowledge. A larger difference existed for the HR website condition, where the higher knowledge participants likely benefited from the extra information available on the website. However, those with lower knowledge had more difficulties in finding information and making decisions. For example, 1 participant commented that they felt that information on the website was repetitive and that it was hard to understand where they needed to go:

I would say it’s a bit cumbersome, and it creates confusion. But, you know, if you search carefully, you are able to find some useful information, but it takes time and effort. I would say, you know, this website assistant can be greatly simplified, and there’s a lot of information redundancy [...] I really need someone, you know, who really knows it, who can provide me with some advice.

ID02, HR website

For ease of use, both higher and lower knowledge participants rated the VBC system as easy to use. The VBC system ratings were also higher than those of the HR website. Individuals with lower health insurance knowledge rated the HR website as more difficult to use than those with higher pre-existing knowledge. This was likely due to differences in how the 2 groups navigated and searched for information. Participants with lower health insurance knowledge tended to describe their search process as reading through the different options laid out on each page before deciding on which link to click on next (eg, “I started with the [State health insurance webpage], just because it’s on top, reading to find clickable items, hit my benefits, and scrolling down to just kind of see what this page had.” [ID11, HR website]). However, even higher knowledge participants noted issues with the organization of the information and knowing where to go next (eg, “To me, it’s just not obvious. I am not sure I need to be clicking multiple times to get where I need to go. I feel like on the very first page I should be able to click health insurance plans.” [ID07, HR website]).

Overall, these findings agree with the other measures; both the guided VBC system and the self-directed HR website were seen as useful and had positive benefits to users. However, the guided VBC system experience was perceived to be easier to use than the self-directed HR website, particularly for participants who had lower pre-experiment health insurance knowledge.

## Discussion

### Principal Findings

This study examined how individuals use 2 different types of health insurance decision aids to make enrollment decisions: (1) a guided VBC system that walked users through factors that are important in health insurance decision-making and provide support for terminology and definitions and (2) a more traditional digital source of health insurance information, an HR website that provides educational information and brochures but requires self-directed navigation through the system. We contrasted these 2 decision aid systems on measures that may impact decision quality: health insurance knowledge, literacy, decision factors, decision conflict, and usability.

The results showed that both types of health insurance decision aids had positive benefits for health insurance knowledge and literacy. Previous health insurance knowledge played an important role in how individuals used the 2 health information decision aids. Individuals with lower pre-experiment knowledge test scores focused on different decision factors and were more conflicted about their final enrollment decisions than those with higher knowledge test scores. Furthermore, although both decision aids resulted in similar changes in the HILM and knowledge test scores, differences exist for the usefulness and ease of use of the 2 systems. HR website navigation was not intuitive, and it took longer to locate information, although users appreciated that it had more details; the VBC system was easier to use but had limited details with some users, indicating that the HR website was still needed as a supplementary companion. Lower knowledge participants, in particular, found the HR website to be less useful and harder to use than those with higher health insurance knowledge. Finally, decision aid tools, such as out-of-pocket cost estimation tools, can lead to confusion when they do not highlight which factors of each plan contribute to the cost estimate. Users wanted a more robust tool that showed the cost breakdown and could help them explore how their use estimates influence cost estimation.

### Comparison With Previous Work

One surprising finding in this study was that the VBC and the HR website had similar effects on health insurance knowledge and literacy. This contrasts with the results of Politi et al [10], who compared a custom-built decision aid (consisting of education, cost estimations, and recommendations) with a traditional government website. Their decision aid resulted in higher literacy and knowledge than websites. This difference could be due to the smaller sample size (n=16 vs n=328) or participant demographics. In addition, our study participants had employer-provided health insurance, whereas the majority in the study by Politi et al [10] were uninsured. A study by Vardell [21] on new employees choosing health insurance found that first-time decision-makers tended to have lower HILM than other participants. Owing to our participants’ previous experience with the plans, they may have had less ability to benefit from a health insurance decision aid. Furthermore, participants in both conditions used similar decision factors, even though the VBC system provided more guidance about the factors to consider. Future work will be required to expand our exploratory study to a more diverse population.

However, interacting with either system appeared to have positive benefits for measures that may lead to more informed decision-making. Participants scored better on the knowledge test and felt more confident about choosing and using their plans (2 subcomponents of health literacy) after using the decision aids. These are positive outcomes given the low health insurance literacy and health insurance plan selection issues previously found in the literature [2,5,21-23]. However, 1 trend in both the literacy measure and the think-aloud interviews was confused about comparing plans. Our participants had difficulty understanding what made the plans unique. This suggests that current decision aids fail to help users develop a mental model of how each plan works. Mental models are a type of internal representation that helps simulate different future outcomes [24,25]. More fully developed mental models may help users explore possible future scenarios and better understand each plan’s strengths and weaknesses. Interestingly, none of our participants used the cost estimation tool to explore these possibilities. Decision aids may require additional guidance for users to explore *edge-cases* rather than just focusing on the most likely scenarios. This type of support is likely more challenging to implement in a self-directed system such as a webpage than a guided VBC decision aid and should be explored in future work.

Finally, the results suggest that pre-existing knowledge may have one of the most significant impacts on the decision-making process regardless of the type of decision aid provided. In our study, participants with higher pre-experiment knowledge test scores had fewer decision conflicts. They also focused on essential decision factors, such as the plan’s ease of use, in contrast to those with lower knowledge test scores, who focused on how easy it was to understand a plan. Our findings agree with previous research indicating that individuals with low HILM scores or limited experience with health insurance decision-making (eg, new employees or young adults) will struggle with the decision process, how to interact with the system, and are more likely to make mistakes when choosing coverage [21,22,26]. Our results also suggest that the unguided nature of the HR website made it more difficult for those with low health insurance knowledge to use and benefit from because they do not have a strategy for searching for relevant information and combining this information together to make a final decision. The VBC, on the other hand, had similar benefits for both low and high knowledge participants, with the main drawback being the lack of detailed information. Thus, VBCs may fulfill their intended purpose of helping those with low health insurance literacy but should be used with a variety of different sources of health insurance information.

### Limitations

A few limitations exist that may impact the generalizability of the results of this study. The study had a small sample of only 16 participants, as recruitment for in-person human subject experiments was impacted by COVID-19. Although these participants generated a large set of think-aloud interview data (approximately 4 hours of audio recordings), the small sample size for the knowledge test and literacy measures made it difficult to compare the VBC and HR websites using inferential statistical analysis. Instead, our analysis focused on interview data and an exploratory analysis of the trends in the questionnaire data. Furthermore, the employees in our sample were full-time employees at a local state university. These included both staff and faculty and may not be fully representative of employees at other types of employers in terms of demographics, health insurance knowledge, and experience with technology. Future work should examine how differences between employee characteristics at different employers may impact user interactions with health insurance decision aids.

### Conclusions

In conclusion, this study showed that health insurance decision aids help individuals improve their knowledge about health insurance and their confidence in selecting and using health insurance plans. In addition, previous health insurance knowledge played a significant role in how users interacted with and benefited from decision aids. Although the study participants indicated that both the VBC and HR website appeared to have a similar effect on these HILMs and decision factors considered, participants perceived the VBC system as easier to use. In contrast, participants with lower prior knowledge appeared to struggle with using the HR website, resulting in lower perceived usefulness and ease of use.

This study’s thematic analysis identified important decision factors among the study participants. Once again, the specific decision aid did not strongly impact the relative importance of the decision factors. However, participants with low health insurance knowledge felt more conflicted about their final mock decisions. In addition, they discussed the health plan’s ease of use and use costs less frequently than others, but they also placed more value on how well the digital aids helped them understand the plan. Finally, more research is required on (1) how decision aids affect mental models of health insurance plans, (2) how decision aids affect user decision strategies and information-seeking strategies, and (3) the development of more robust cost estimation tools that help users differentiate plans for *edge-cases* and out-of-pocket costs.

## Abbreviations

ETA: Emergent Themes Analysis
HILM: Health Insurance Literacy Measure
HR: human resources
SURE: Sure of myself, Understand information, Risk-benefit ratio, Encouragement
VBC: virtual benefits counselor
